# Supplementary material for: Atlas of plasma NMR biomarkers for health and disease in 118,461 individuals from the UK Biobank
Source: Nat Commun. 2023 Feb 3;14:604. doi: 10.1038/s41467-023-36231-7 (PMC9898515; doi:10.1038/s41467-023-36231-7)
Supplement: Supplementary file 1 — Supplementary Information [file 41467_2023_36231_MOESM1_ESM.pdf]

Atlas of plasma NMR biomarkers for health and disease in 118,461 individuals from the UK Biobank

Julkunen *et al.*

Supplementary Methods

Nightingale Health NMR biomarker profiling of UK Biobank samples

Quality control protocol

Pre-specified metrics on the biomarker consistency were agreed between UK Biobank and Nightingale Health to ensure the quality of results throughout the project. The full project was initiated after the consistency metrics of the pilot were met. Two internal control samples provided by Nightingale Health were included in each 96-well plate for tracking the consistency across multiple spectrometers during the project. Four sets of internal control samples with different biomarker concentration span were used across the 1,352 96-well plates measured. These were interleaved between the NMR instruments for extended periods of the project duration. An example of such continuous quality control is illustrated in Supplementary Figure 3 for the case of leucine.

Technical and biological repeatability

Two blind duplicate samples provided by UK Biobank were included on each 96-well plate. The position information of these blind duplicates was revealed only after interim results delivery to UK Biobank. Supplementary Figure 4 illustrates the distribution of coefficients of variation (CV) across the biomarker measures, both for the UK Biobank's blind duplicates and Nightingale Health's internal control samples. The CVs are below 5% for most the biomarkers in both instances. These results fulfilled the pre-specified CV targets across the biomarker measures for each consecutively measured set of approximately 20,000 samples. Prior studies on smaller scale have also reported representative CVs for blind duplicate samples as well as for repeat control samples for NMR biomarkers from the Nightingale Health platform.[1,2] The technical consistency of measurements over consecutive shipment batches and in different NMR spectrometers are illustrated for all the NMR biomarkers in the UK Biobank data resource (https://biobank.ndph.ox.ac.uk/showcase/label.cgi?id=220). This resource also shows the correlation of blinded duplicate samples for each biomarker, as well as the biological consistency in repeat-visit samples drawn from the same individuals four years apart.

Quality control flags

The Nightingale Health NMR platform involves integrated quality procedures to report signs of degradation and contamination issues in each plasma sample. These are reported as flags along with the biomarker concentration result data. Issues affecting the whole sample are reported as sample-level flags; issues affecting only certain biomarkers are reported as biomarker-level flags, provided as a separate data field for each biomarker. In general, if a biomarker has a flag but the value is still provided, it indicates that the presence of the interfering substance is low and deemed not to interfere with the quantification of the biomarker (i.e., the value can be trusted). There is no need to a priori remove any biomarker values based on the flags; however, researchers may consider performing sensitivity analyses as described in the section "Recommended approaches for data pre-processing and epidemiological analyses".

Technical variation and outlier plates

An independent study from Cambridge University conducted post-measurement quality control and analysed sources of technical variation in the NMR biomarker data[6]. This study identified spectrometer used and time from plasma preparation to measurement as the only

two notable sources of variation. Each factor explained 1-3% of variation for the majority number of biomarkers, and only the amino acids histidine and alanine had substantially higher for technical variation. Researchers may therefore consider regressing out or adjusting for spectrometer as a factor in epidemiological analyses.

The same study highlighted a small number of outlier plates with deviating concentration across many biomarkers[6]. This technical variation was deemed to arise from UK Biobank's sample plating process. A median of 9 outlier plates were identified across the biomarkers, with a maximum of 20 outlier plates for albumin. The authors recommended to remove data from these outlier plates; however, with only ~1% of the samples affected the impact on epidemiological associations is modest. An R package has been made available to remove the technical variation, including the outlier plates. This can especially provide slight power gains for genome-wide association analyses, whereas the impact on biomarker-disease associations is minor.

Recommended data processing for epidemiological analyses

The NMR biomarker data in the UK Biobank can generally be used for epidemiological analyses without any preprocessing and can in principle be analysed in the same manner as the clinical chemistry data available in UK Biobank. The clinical chemistry data, already available in the full cohort, can also be used as a positive control in case of overlapping measures, and as means to put association magnitudes into context of established clinical measures. The approach of 4SD outlier exclusion and log-transformation chosen for the present study is applied to have a consistent approach, but omission or variations of these steps generally has minute influence on the biomarker-disease associations.

The degree of missingness of any biomarker is generally small (<1%). For analyses of samples marked as zero concentration value we recommend replacing the zero values with the values just below the lowest observed value, since it avoids artificial drops in the distributions. For analyses that require complete data of multiple or all biomarkers simultaneously, we recommend methods for imputation rather than excluding the entire sample if there are a few missing biomarkers.

Accounting for quality control flags

Biomarker values substantially affected by interfering substances have been removed during the quality control procedures. However, researchers may consider performing sensitivity analyses by excluding samples flagged with "Low protein", which may indicate more severe sample dilution. Biomarker values flagged with "Below limit of quantification" may also be omitted in sensitivity analyses, since this flag indicates that the concentration of the given biomarker is smaller than the range where the quantification of the NMR biomarkers is considered highly accurate. The analyses done for the biomarker disease-atlas does not omit these values.

Comparison to other multi-biomarker platforms in smaller cohorts

The biomarker coverage from the Nightingale Health NMR platform is mostly distinct from those of mass-spectrometry based metabolomics assays[3,4]. Less than 20 out the 249 biomarkers are quantified by the main mass-spectrometry metabolomics vendors. Only in the case of amino acids and glycolysis metabolites is there direct overlap with mass spectrometry platforms. The main reason for the limited biomarker overlap is that mass-spectrometry platforms are generally not able to quantify the detailed lipoprotein measures obtained by the Nightingale Health NMR platform, since the physiological character of lipoprotein particles are

destroyed in mass spectrometry. Furthermore, important analytes not commonly analyzed by mass spectrometry include the GlycA composite-protein biomarker as well as aggregate fatty acid measures, such as omega-3%, which are relevant for dietary studies and supplementation trials and often more interpretable than molecule-specific fatty acids.

The measurements of fatty acids, amino acids and glycolysis metabolites by the Nightingale Health NMR platform have been certified for clinical use. To further demonstrate the validity of NMR biomarker quantification, we report previously unpublished correlations of amino acids, glycolysis metabolites and circulating fatty acids measured with three other analytical platforms. We note that these measurements were not done at the same time from split aliquots, and therefore do not represent strict analytical comparisons but rather consistency in cohort settings, potentially from different blood specimens and measured years apart.

Supplementary Figure 7 shows scatter plots of absolute and relative fatty acids measured by Nightingale Health NMR platform in comparison to gas chromatography (Vitas Analytical Services, Oslo, Norway). The samples are from a familial hypercholesterolemia cohort of n =144 individuals[9]. The correlations were particularly high for absolute fatty acid measures (r = 0.89-0.98) and slightly lower for fatty acid ratios, relative to total fatty acids (r = 0.80-0.92). These results are consistent with comparisons of NMR with gas chromatography fatty acids, using a prior version of the Nightingale Health biomarker platform involving a lipid extraction step[2].

Supplementary Figure 8 shows scatter plots of amino acids in comparison to the Biocrates p180 mass spectrometry platform (Innsbruck, Austria) in the ADNI1 cohort (n = 749). Correlations were highest for branched-chain and aromatic amino acids as well as alanine and glycine (r = 0.78-0.90) and lower for glutamine (r =0.65) and histidine (r = 0.54). Supplementary Figure 8 also shows ascatter plot for the ketone body 3-hydroxybutyrate measured by NMR, in comparison to a cyclic enzymatic method (Wako Chemicals GmbH, Neuss, Germany) in an Italian cohort[10]. The correlation with NMR-based measure was r = 0.98. From the same study, the triglyceride-rich lipoprotein cholesterol measured by the Nightingale Health NMR platform has previously been reported to correlate well with ultra-centrifugation (r = 0.90)[11].

Finally, Pearson's correlations of amino acids and glycolysis-related metabolites measured by the Nightingale Health platform and the Metabolon HD4 mass-spectrometry platform (Morrisville, North Carolina, US) from the same samples were the following in the Qatar Metabolomics Study on Diabetes cohort (QMDiab): leucine 0.86; valine 0.82; phenylalanine 0.67; tyrosine 0.90; glutamine 0.75; histidine 0.62; alanine 0.75; glucose 0.86; lactate 0.93; citrate 0.81 (results courtesy of Karsten Suhre, Weill Cornell Medicine Qatar). These results are consistent with other studies reporting the medium to high consistency (r = 0.42-0.85) of the few biomarkers overlapping between Nightingale Health NMR and mass-spectrometry data from Metabolon and Biocrates[12,13].

References

1. Holmes, M. V. *et al.* Lipids, Lipoproteins, and Metabolites and Risk of Myocardial Infarction and Stroke. *J. Am. Coll. Cardiol.* **71**, 620–632 (2018).
2. Kettunen, J. *et al.* Genome-wide study for circulating metabolites identifies 62 loci and reveals novel systemic effects of LPA. *Nat. Commun.* **7**, 11122 (2016)..
3. Soininen, P., Kangas, A. J., Würtz, P., Suna, T. & Ala-Korpela, M. Quantitative Serum Nuclear Magnetic Resonance Metabolomics in Cardiovascular Epidemiology and Genetics. *Circ. Cardiovasc. Genet.* **8**, 192–206 (2015).
4. Würtz, P. *et al.* Quantitative Serum Nuclear Magnetic Resonance Metabolomics in Large-Scale Epidemiology: A Primer on -Omic Technologies. *Am. J. Epidemiol.* **186**, 1084–1096 (2017)
5. Allen, N. E. *et al.* Approaches to minimising the epidemiological impact of sources of systematic and random variation that may affect biochemistry assay data in UK Biobank. *Wellcome Open Res.* **5**, 222 (2021).
6. Ritchie, S. C. *et al. Quality control and removal of technical variation of NMR metabolic biomarker data in ~120,000 UK Biobank participants*. http://medrxiv.org/lookup/doi/10.1101/2021.09.24.21264079 (2021) doi:10.1101/2021.09.24.21264079.
7. Borodulin, K. *et al.* Cohort Profile: The National FINRISK Study. *Int. J. Epidemiol.* **47**, 696–696i (2018).
8. Tikkanen, E. *et al.* Metabolic Biomarker Discovery for Risk of Peripheral Artery Disease Compared With Coronary Artery Disease: Lipoprotein and Metabolite Profiling of 31 657 Individuals From 5 Prospective Cohorts. *J. Am. Heart Assoc.* **10**, e021995 (2021).
9. Øyri, L. K. L. *et al.* Delayed postprandial TAG peak after intake of SFA compared with PUFA in subjects with and without familial hypercholesterolaemia: a randomised controlled trial. *Br. J. Nutr.* **119**, 1142–1150 (2018).
10. Tikkanen, E. *et al.* Metabolomic Signature of Angiopoietin-Like Protein 3 Deficiency in Fasting and Postprandial State. *Arterioscler. Thromb. Vasc. Biol.* **39**, 665–674 (2019).
11. Würtz, P. & Soininen, P. Reply to: "Methodological issues regarding: 'A third of nonfasting plasma cholesterol is in remnant lipoproteins: Lipoprotein subclass profiling in 9293 individuals'". *Atherosclerosis* **302**, 59–61 (2020).
12. Deelen, J. *et al.* A metabolic profile of all-cause mortality risk identified in an observational study of 44,168 individuals. *Nat. Commun.* **10**, 3346 (2019).
13. Schmidt, J. A. *et al.* NMR Metabolite Profiles in Male Meat-Eaters, Fish-Eaters, Vegetarians and Vegans, and Comparison with MS Metabolite Profiles. *Metabolites* **11**, 121 (2021).
14. Holmes, M. V. & Ala-Korpela, M. What is 'LDL cholesterol'? *Nat. Rev. Cardiol.* **16**, 197–198 (2019).

Supplementary Table 1. Baseline characteristics and event numbers in the five Finnish cohorts included in the replication analyses.

	FINRISK 1997	FINRISK 2002	FINRISK 2007	FINRISK 2012	Health 2000
Number of participants	7580	7917	5966	5516	7100
Age (median, [range])	49 [25-74]	49 [25-74]	52 [25-74]	53 [25-74]	53 [30-98]
Females (%)	50	55	53	52	55
Smoking prevalence (%)	23.4	25.5	20.4	19.0	20.4
Fasting time, mean (h)	6.0	5.8	5.3	5.8	2.9
Self-reported cholesterol lowering medication use (%)	3.5	7.1	14.2	15.6	5.7
Number of events:					
All-cause mortality	1335	673	303	78	1540
Atrial fibrillation	717	451	230	92	627
Cancer mortality	448	236	110	38	440
Chronic kidney failure	102	63	34	6	123
COPD	258	178	73	33	192
Diabetes	895	675	324	98	712
Fibrosis and cirrhosis of the liver	16	16	6	1	24
Heart disease mortality	466	218	88	16	558
Heart failure	1151	645	348	103	733
Liver diseases	124	109	39	16	129
Lung cancer	109	49	19	8	22
Major adverse cardiovascular event	1691	1014	543	186	1207
Myocardial infarction	447	254	123	48	428
Rheuma	372	289	124	51	251

Supplementary Table 2. Endpoint definitions for the replication analyses.

Pre-defined endpoint	ICD-10 codes used for endpoint definition in THL Biobank analyses	ICD-10 codes used for UK Biobank analyses
Major adverse cardiovascular event	I21-22, I50, I61, I63-64, I20.0, I11.0, I13.0, I13.2<br>Death records: I46, R96, R98	I21-22, I50, I61, I63-64
Diabetes	E10-E14*	E10-E14
COPD	J43-J44	J43-J44
Chronic kidney failure	N18-N19	N18-N19
Liver diseases	K70-K77	K70-K77
Myocardial infarction	I21-22	I21-22
Heart failure	I50, I11.0, I13.0, I13.2	I50
Atrial fibrillation	I48	I48
Lung cancer	Lung cancer in cancer register	C34
Fibrosis and cirrhosis of the liver	K74	K74
Rheumatoid disease	M05-M13, M32, M33, M45	M05-M13, M32, M33, M45
Heart disease mortality	I20-I25<br>Death records: I46, R96, R98	I20-25 (death records)
Cancer mortality	Any cancer in cancer register	C00-C99 (death records)

* Type 1 or type 2 diabetes; also national reimbursement records for anti-diabetic medication were use.

Supplementary Table 3. Endpoints considered for sex-specific association analyses.

Sex	ICD-10 codes
Female	C50 Malignant neoplasm of breast<br>C51-C58 Malignant neoplasms of female genital organs<br>D05 Carcinoma in situ of breast<br>D06 Carcinoma in situ of cervix uteri<br>D25 Leiomyoma of uterus<br>D26 Other benign neoplasms of uterus<br>D27 Benign neoplasm of ovary<br>D28 Benign neoplasm of other and unspecified female genital organs<br>D39 Neoplasm of uncertain or unknown behaviour of female genital organs<br>N60-N64 Disorders of breast<br>N70-N77 Inflammatory diseases of female pelvic organs<br>N80-N98 Noninflammatory disorders of female genital tract<br>O00-O99 Pregnancy, childbirth and the puerperium
Male	N40-N51 Diseases of male genital organs<br>C60-C63 Malignant neoplasms of male genital organs<br>D29 Benign neoplasm of male genital organs<br>D40 Neoplasm of uncertain or unknown behaviour of male genital organs

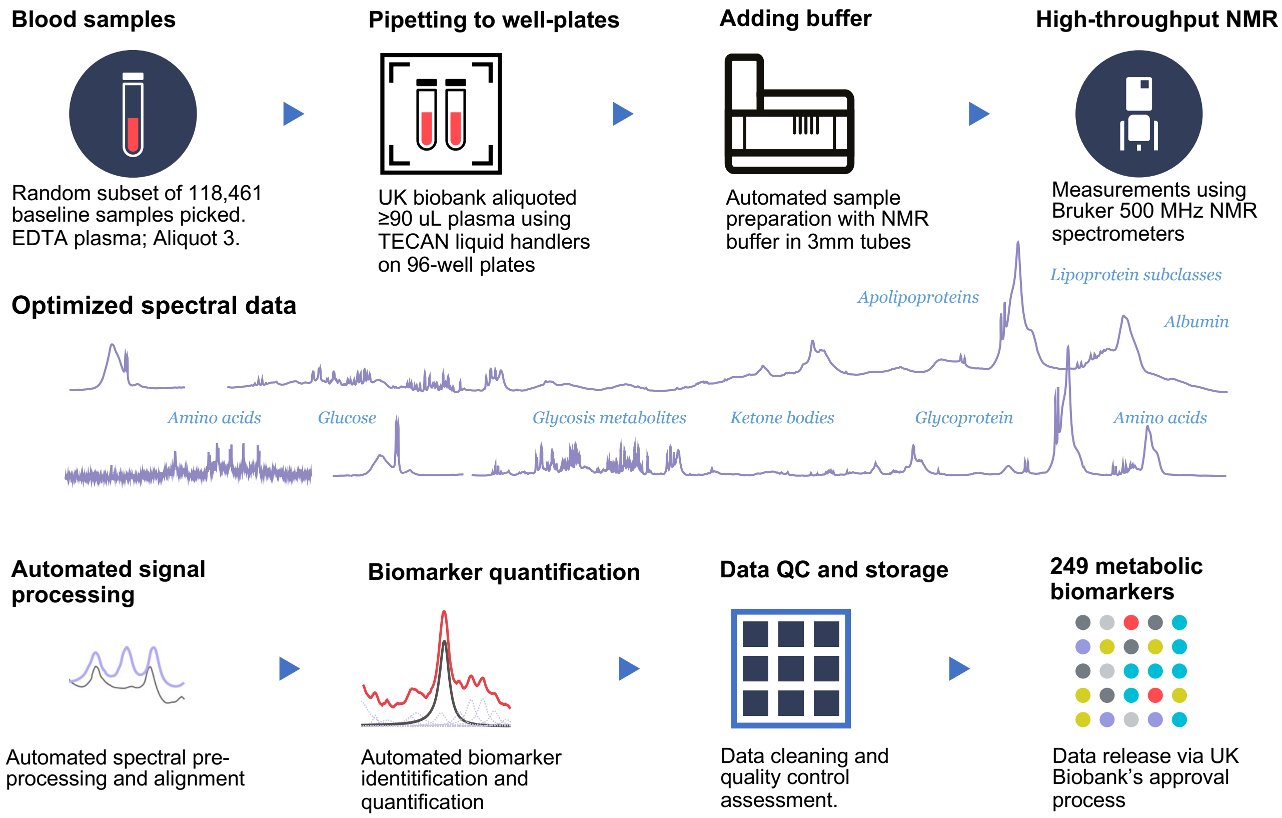

Supplementary Figure 1. Key steps of the biomarker measurement process in the Nightingale Health UK Biobank initiative.

Amino acids (mmol/l)
* Alanine mmol/l
Glutamine mmol/l
* Glycine mmol/l
* Histidine mmol/l
* Phenylalanine mmol/l
* Tyrosine mmol/l
* Isoleucine mmol/l
* Leucine mmol/l
* Valine mmol/l
* Total branched-chain amino acids

Glycolysis related metabolites (mmol/l)
* Glucose
* Lactate
Pyruvate
Citrate

Ketone bodies (mmol/l)
3-hydroxybutyrate
Acetoacetate
Acetone
Acetate

Inflammation (mmol/l)
* Glycoprotein acetyls (GlycA)

Fluid balance (mmol/l)
* Creatinine
* Albumin

Fatty acids (mmol/l)
* Total fatty acids
* Omega-3 fatty acids
* Omega-6 fatty acids
* Polyunsaturated fatty acids (PUFA)
* Monounsaturated fatty acids (MUFA)
* Saturated fatty acids
Linoleic acid
* Docosahexaenoic acid (DHA)

Fatty acid ratios (%)
* Omega-3 fatty acids ratio to total fatty acids
* Omega-6 fatty acids ratio to total fatty acids
* PUFA ratio to total fatty acids
* MUFA ratio to total fatty acids
* Saturated fatty acids ratio to total fatty acids
Linoleic acid ratio to total fatty acids
* DHA ratio to total fatty acids
* PUFA to MUFA ratio
* Omega-6 fatty acids to omega-3 fatty acids ratio
Degree of unsaturation

Other lipids (mmol/l)
Phosphoglycerides
Ratio of triglycerides to phosphoglycerides ratio
Total cholines
Phosphatidylcholines
Sphingomyelins

Cholesterol (mmol/l)
* Total cholesterol
Non-HDL-C
Remnant cholesterol
* VLDL cholesterol
* Clinical LDL cholesterol
LDL cholesterol (size-specific)
* HDL cholesterol

Triglycerides (mmol/l)
* Total triglycerides
Triglycerides in VLDL
Triglycerides in LDL
Triglycerides in HDL

Apolipoproteins (g/l)
* Apolipoprotein B
* Apolipoprotein A1 g/l
* Apolipoprotein B to apolipoprotein A1 ratio

Lipoprotein particle size (nm)
Average diameter for VLDL particles
Average diameter for LDL particles
Average diameter for HDL particles

Lipoprotein particle concentrations (mmol/l)
Total concentration of lipoprotein particles
Concentration of VLDL particles
Concentration of LDL particles
Concentration of HDL particles

Total lipids in lipoprotein particles (mmol/l)
Total lipids in lipoprotein particles
Total lipids in VLDL
Total lipids in LDL
Total lipids in HDL mmol/l

Phospholipids (mmol/l)
Total phospholipids
Phospholipids in VLDL
Phospholipids in LDL
Phospholipids in HDL

Cholesteryl esters (mmol/l)
Total esterified cholesterol
Cholesteryl esters in VLDL
Cholesteryl esters in LDL
Cholesteryl esters in HDL

Free cholesterol (mmol/l)
Total free cholesterol
Free cholesterol in VLDL
Free cholesterol in LDL
Free cholesterol in HDL

Particle concentration and lipid composition for 14 lipoprotein subclasses
Particle concentration (mmol/l)
Total lipids (mmol/l)
Phospholipids (mmol/l and % of total lipids)
Cholesterol (mmol/l and % of total lipids)
Cholesteryl esters (mmol/l and % of total lipids)
Free cholesterol (mmol/l and % of total lipids)
Triglycerides (mmol/l and % of total lipids)

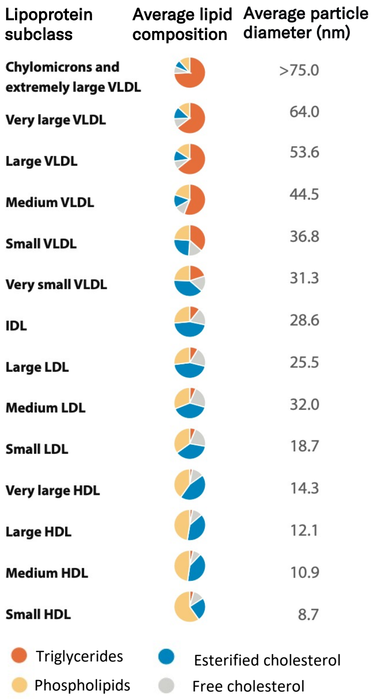

Supplementary Figure 2. Overview of biomarkers quantified by the Nightingale Health NMR platform. Majority of the biomarker measures reflect lipid metabolism, but also cover proteolysis, glycolysis, and ketolysis. The biomarkers reflect diverse health aspects such as chronic inflammation, dietary intake and the risk of various diseases. The 37 biomarkers marked with an asterisk (*) are those currently certified for diagnostics use. Lipoprotein subclasses are defined in particle-size specific manner calibrated against gel permeation high-performance liquid chromatography[11]. The average particle size of each subclass is indicated. For each of the 14 lipoprotein subclasses, 12 measures are provided: the circulating concentration of total lipids in the particles (sum of free and esterified cholesterol, triglycerides and phospholipids), the particle concentration, and the absolute circulating concentration of five main lipids (free, esterified and total cholesterol, triglycerides and phospholipids) and the relative proportions of these five lipids in each particle subclass. The lipoprotein subclasses are defined according to their particle size as illustrated in the figure. HDL indicates high-density lipoprotein; IDL, intermediate-density lipoprotein; LDL, low-density lipoprotein; VLDL, very-low density lipoprotein. 'Clinical LDL cholesterol' and 'size-specific LDL cholesterol' refer to different methods for defining LDL[14]. 'Clinical LDL cholesterol' is the measure that provides concentrations consistent with routine clinical chemistry and the Friedewald equation for LDL-cholesterol.

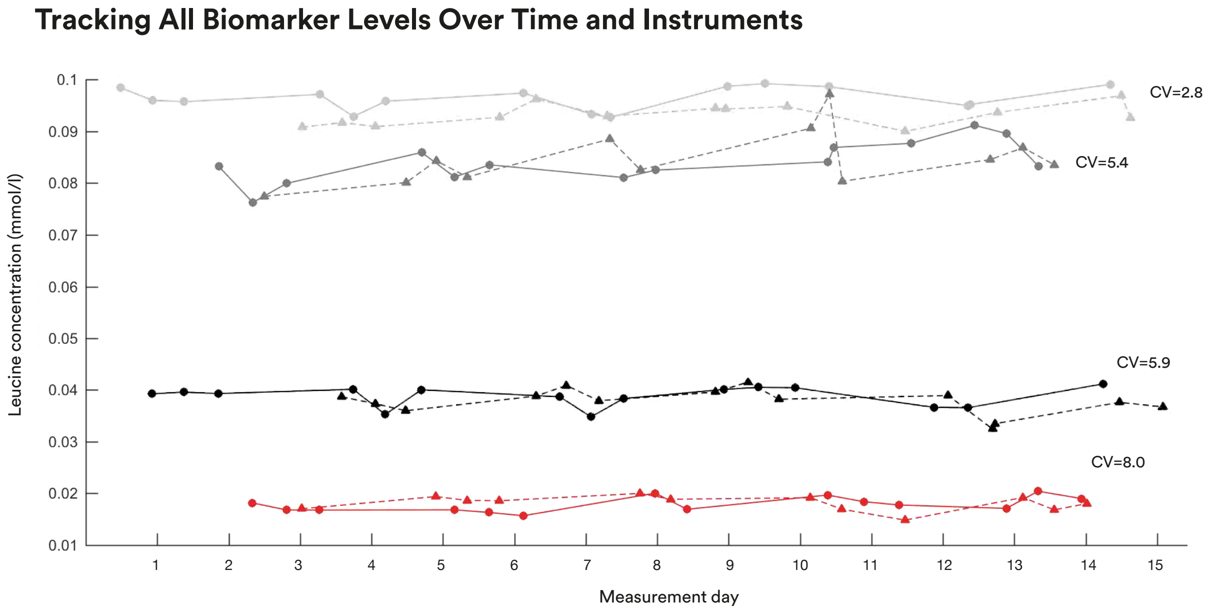

Supplementary Figure 3: Tracking the biomarker quantification along the measurements. The consistency of the biomarker quantification in the control samples is illustrated for leucine; similar tracking was done for all biomarkers. Different colors show results for four control samples that are measured interleaved in NMR instruments during the project course. Dashed and full-blown lines indicate results from two different NMR instruments.

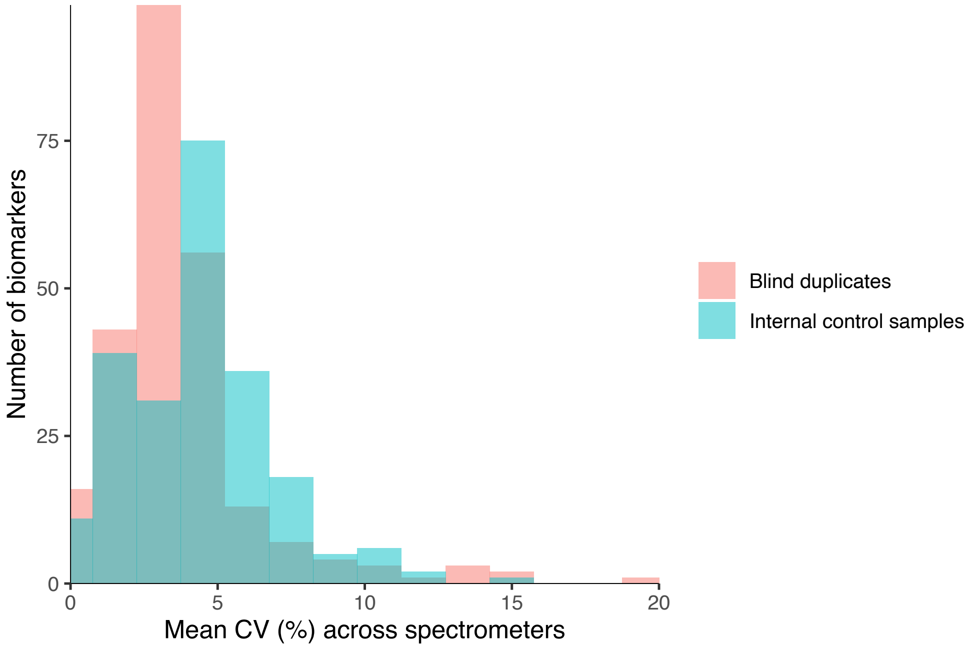

Supplementary Figure 4. Distributions of coefficients of variation (CV) for the 249 metabolic measures. Results for UK Biobank's blind duplicate samples are shown in red and for internal control samples in blue. The CVs are assessed across the six NMR spectrometers used for the measurements. The coefficients of variation for each biomarker is given in the UK Biobank data resource (https://biobank.ndph.ox.ac.uk/showcase/label.cgi?id=220).

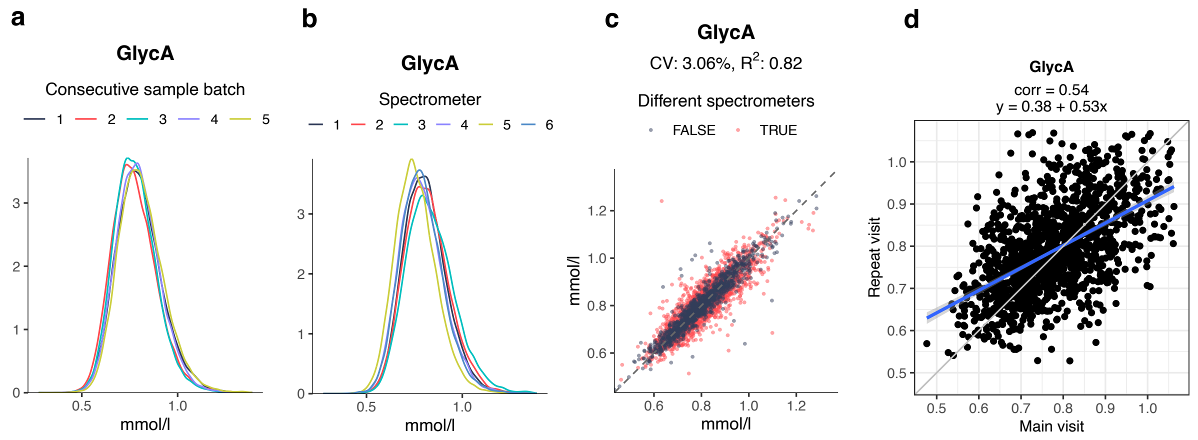

Supplementary Figure 5. Technical and biological repeatability for glycoprotein acetyls (GlycA). Technical consistency in terms of a) distributions of consecutive batches of sample shipments, b) distributions in different spectrometers, c) consistency of ~650 blind duplicates samples (giving rise to a between-instrument CV of 3%). Panel d) shows the biological repeatability for measurements from blood samples from the same individuals drawn ~4 years apart for a approximately 1500 samples. The correponsding plots for each biomarker is given in the UK Biobank data resource (https://biobank.ndph.ox.ac.uk/showcase/label.cgi?id=220).

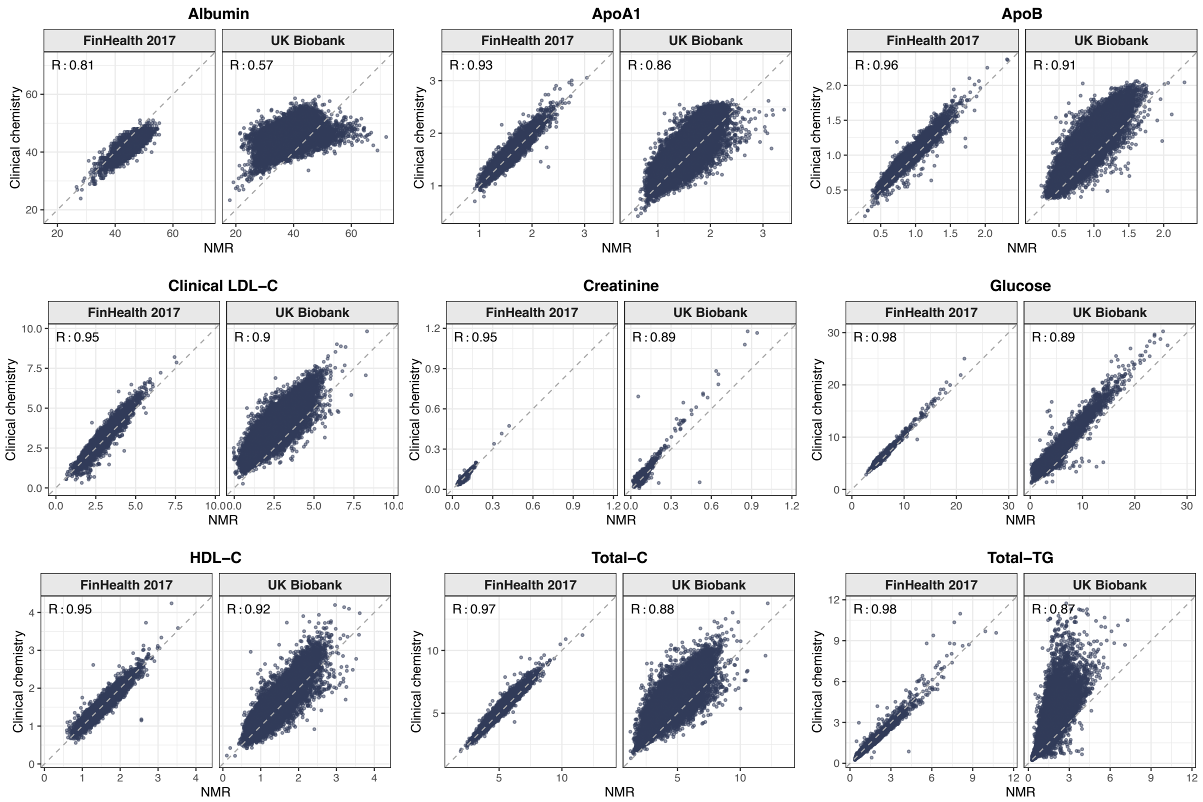

Supplementary Figure 6. Comparisons of the NMR biomarker measurements to routine clinical chemistry. Scatterplots of lipids and other routine biomarkers for which both NMR and clinical chemistry measurements are available in the UK Biobank (n=118,000) and the FinHealth 2017 cohort (n=6,000). Correlation coefficients (R) represent linear Pearson's correlations. The regression line and the corresponding equation represent the slope and offset from ordinary least squares linear regression fit. Clinical chemistry was measured using Beckman Coulter AU5800 instruments. Direct LDL-C was measured by enzymatic selective protection and compared to the corresponding 'clinical LDL-C' measure in the NMR biomarker panel. The more pronounced deviations in correlations and absolute concentrations in UK Biobank compared to FinHealth 2017 samples are primarily due a known dilution issue in the UK biobank samples as described in Supplementary Methods.

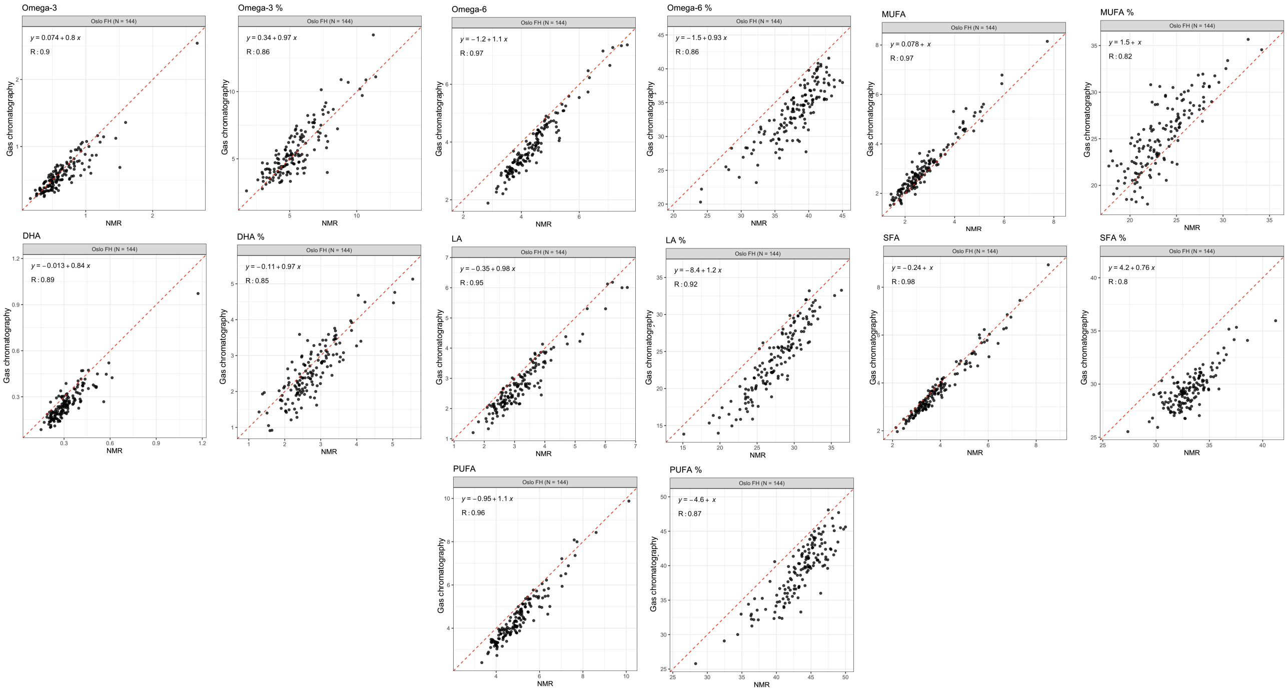

Supplementary Figure 7. Comparisons of the NMR fatty acid biomarker measurements to gas chromatography. Scatter plots of fatty acids quantified with NMR and gas chromatography (n=144; study on familial hypercholesterolemia from University of Oslo, Norway[9]). Correlation coefficients (R) represent linear Pearson's correlations. The regression line and the corresponding equation represent the slope and offset from ordinary least squares linear regression fit.

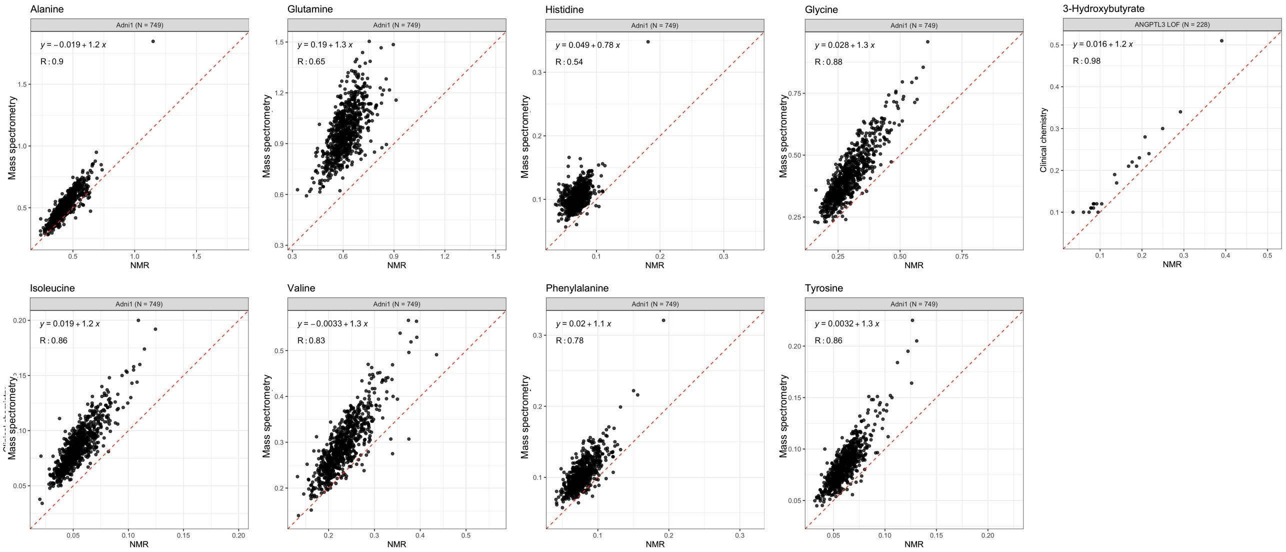

Supplementary Figure 8. Comparisons of the NMR biomarker measurements to mass spectrometry and enzymatic methods. Scatter plots of amino acids quantified with Nightingale Health NMR platform in comparison to Biocrates p180 mass spectrometry in the ADNI1 cohort (n = 749). The plot for 3-hydroxybutyrate shows the comparison to measurements with an enzymatic method in an Italian study of postprandial effects in ANGPTL3 loss-of-function carriers and their controls[10] (n = 228). Correlation coefficients (R) represent linear Pearson's correlations. The regression line and the corresponding equation represent the slope and offset from ordinary least squares linear regression fit.

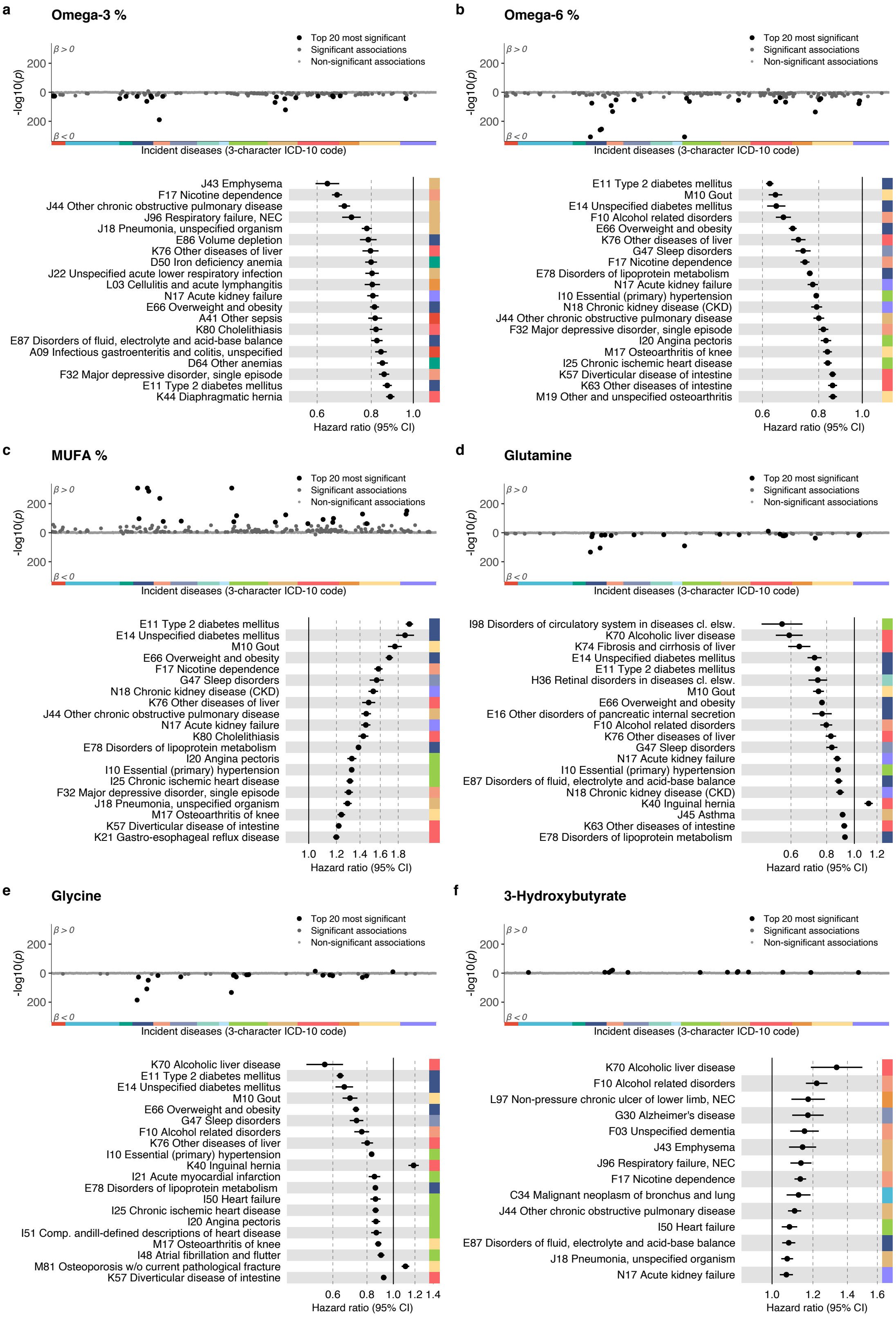

Supplementary Figure 9: Examples of biomarkers for future disease onset. Example of association discovery for six biomarkers: a) Omega-3%, b) Omega-6%, c) Ratio of monounsaturated fatty acids to total fatty acids (MUFA%), d) Glutamine, e) Glycine and f) 3-Hydroxybutyrate. The top panel shows a mirrored Manhattan-style plot of –log transformed p-values with the incidence of diseases with > 50 events across ICD-10 chapters from A to N. Positive associations are displayed on the upper half of the plot, inverse associations on the bottom half. The color coding of indicates distinct ICD-10 chapters, following the color coding in Figure 2. The bottom panel highlights 20 of the most significant associations, arranged according to a decreasing association magnitude. Hazard ratios and 95% confidence intervals (CI) are shown per SD-scaled biomarker concentrations to facilitate comparison. Similar plots for all 249 biomarker measures across all endpoints analysed are available in the biomarker-disease atlas.

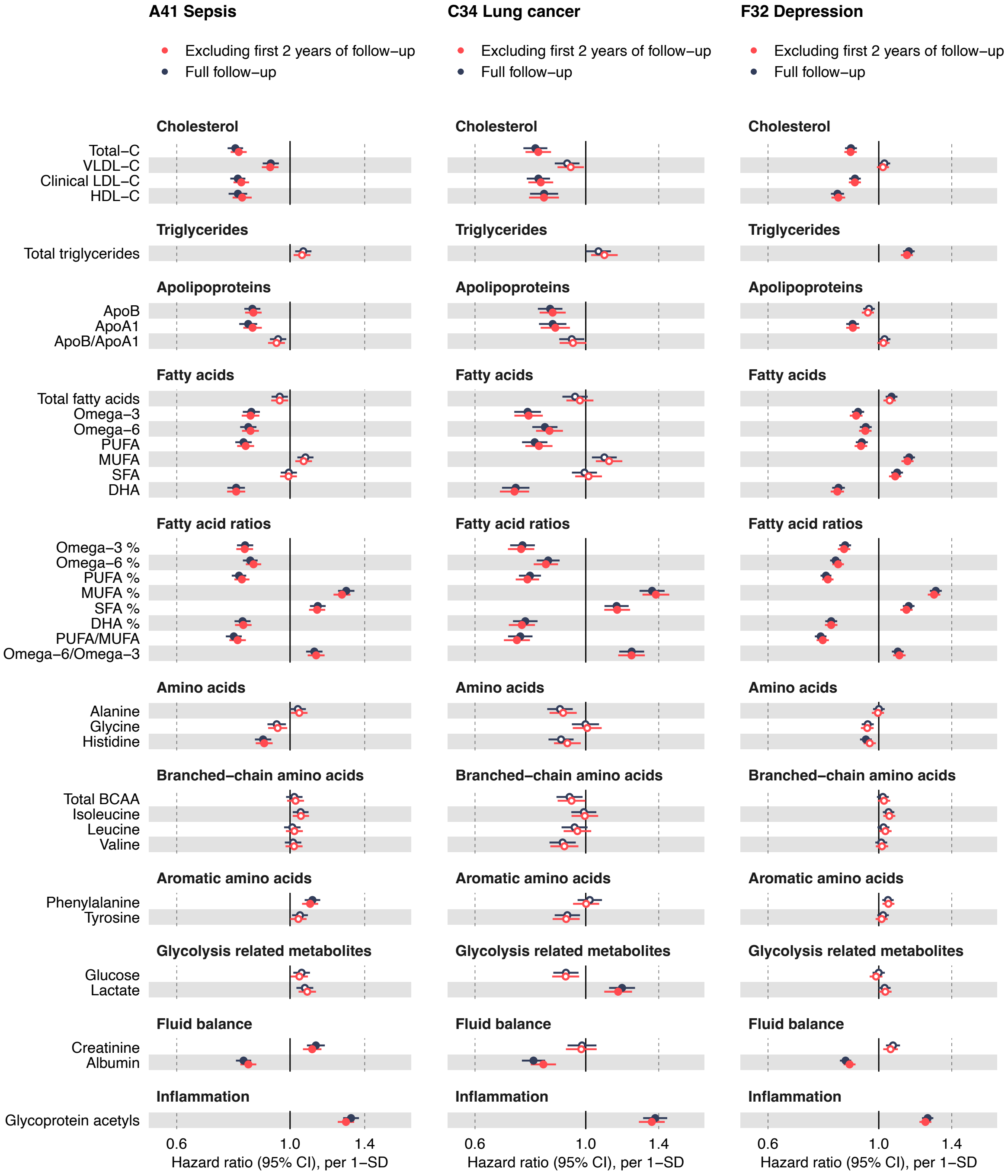
A41 Sepsis
C34 Lung cancer
F32 Depression
Excluding first 2 years of follow−up
Full follow−up
Cholesterol
Total−C
VLDL−C
Clinical LDL−C
HDL−C
Triglycerides
Total triglycerides
Apolipoproteins
ApoB
ApoA1
ApoB/ApoA1
Fatty acids
Total fatty acids
Omega−3
Omega−6
PUFA
MUFA
SFA
DHA
Fatty acid ratios
Omega−3 %
Omega−6 %
PUFA %
MUFA %
SFA %
DHA %
PUFA/MUFA
Omega−6/Omega−3
Amino acids
Alanine
Glycine
Histidine
Branched−chain amino acids
Total BCAA
Isoleucine
Leucine
Valine
Aromatic amino acids
Phenylalanine
Tyrosine
Glycolysis related metabolites
Glucose
Lactate
Fluid balance
Creatinine
Albumin
Inflammation
Glycoprotein acetyls
0.6
1.0
1.4
Hazard ratio (95% CI), per 1−SD

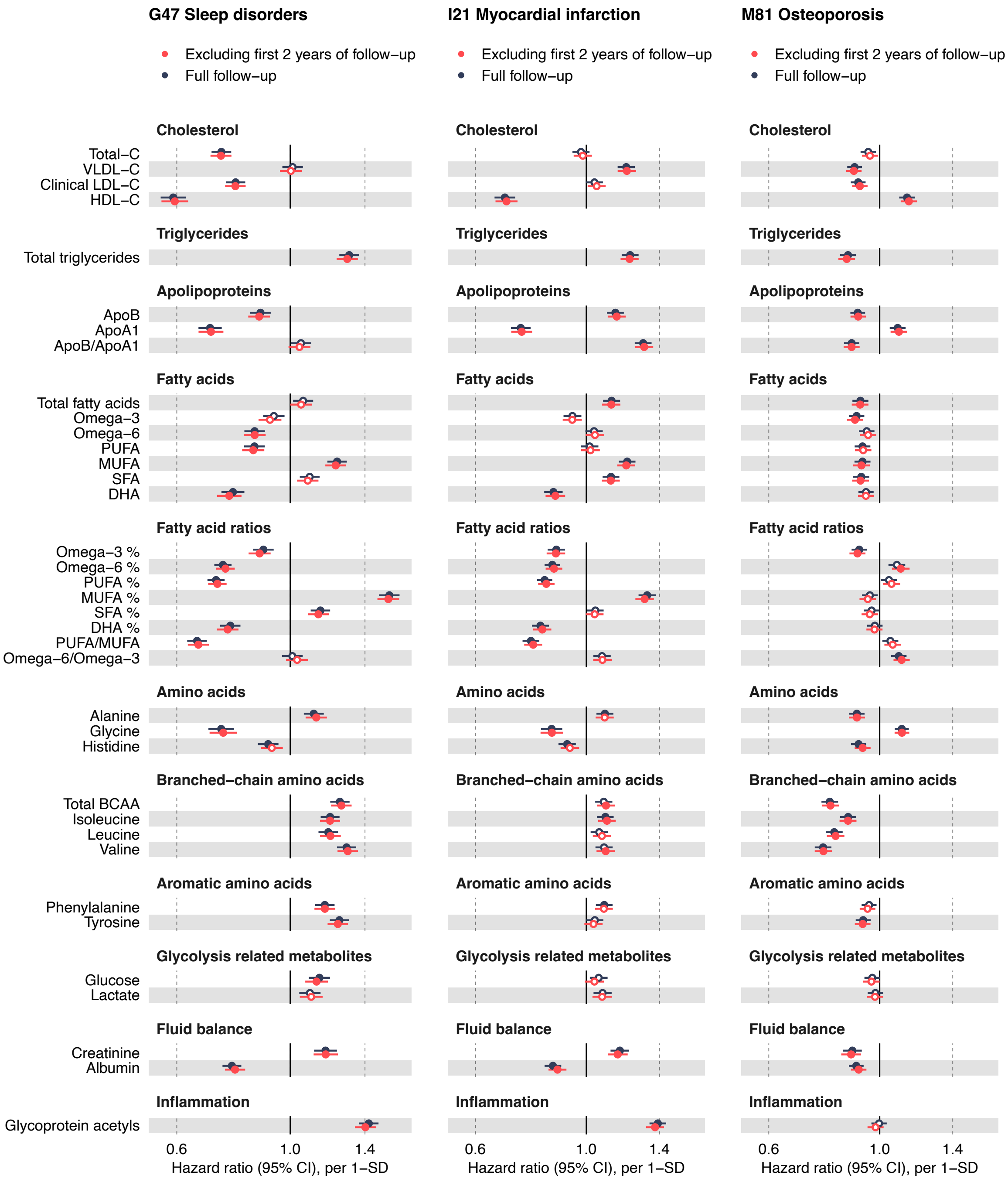

Supplementary Figure 10: Biomarker association profiles from a sensitivity analysis excluding the first two years of follow-up. Hazard ratios of 37 biomarkers with the incidence of six disease examples from a sensitivity analysis excluding the first two years of follow-up (red) in comparison to the full follow-up (black). Hazard ratios and 95% confidence intervals (CI) are shown per SD units. The models were adjusted for age, sex and UK biobank assessment center, using age as the timescale of the Cox proportional hazards regression. Filled points indicate statistically significant associations ($p < 5e\text{-}5$), and hollow points non-significant ones. BCAA indicates branched-chain amino acids; DHA: docosahexaenoic acid; MUFA: monounsaturated fatty acids; PUFA: polyunsaturated fatty acids; SFA: saturated fatty acids.

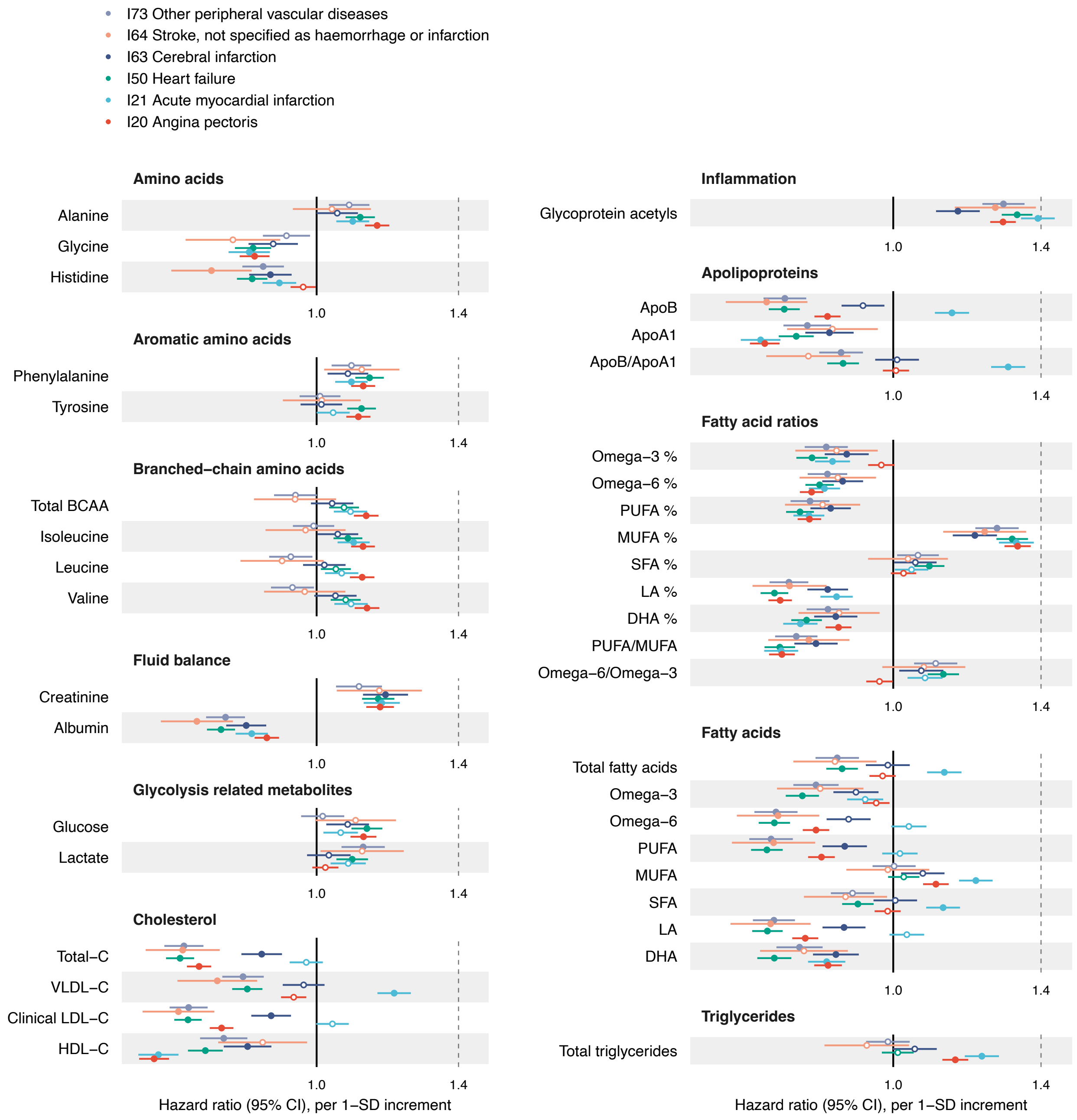

Supplementary Figure 11. Biomarker profiles for incidence of cardiovascular diseases. Hazard ratios of biomarkers with the incidence of six cardiovascular disease endpoints: I20 Angina pectoris (red; n = 115 154, 4 502 events), I21 Acute myocardial infarction (light blue; n = 116 781, 2 523 events), I50 Heart failure (green; n = 117 498, 3 150 events), I63 Cerebral infarction (dark blue; n = 117 724, 1 608 events), I64 Stroke, not specified as haemorrhage or infarction (orange; n = 117 865, 456 events) and I73 Other peripheral vascular disease (lavender; n = 117 597, 1 666 events). The biomarkers represent 37 clinically validated biomarkers in the Nightingale NMR platform. Hazard ratios and 95% confidence intervals (CI) are shown per SD-scaled biomarker concentrations to facilitate comparison. The models were adjusted for age, sex and UK biobank assessment center, using age as the timescale of the Cox proportional hazards regression. Filled points indicate statistically significant ($p < 5e-5$) associations, hollow points non-significant ones. BCAA indicates branched-chain amino acids; DHA: docosahexaenoic acid; MUFA: monounsaturated fatty acids; PUFA: polyunsaturated fatty acids; SFA: saturated fatty acids.

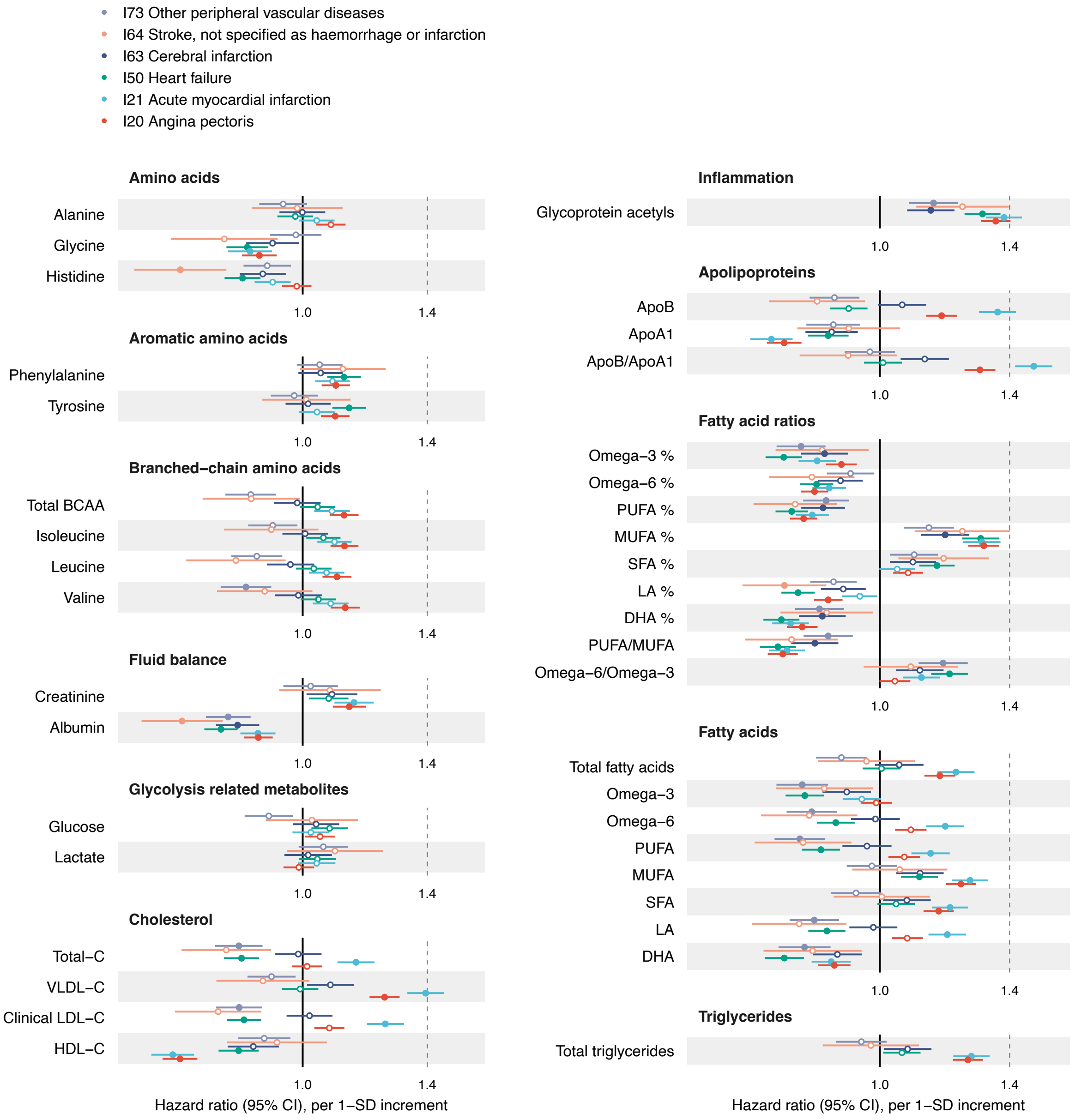

Supplementary Figure 12. Biomarker profiles for incidence of cardiovascular diseases in a population free of cholesterol lowering medication. Hazard ratios of biomarkers with the incidence of six cardiovascular disease endpoints: I20 Angina pectoris (red; n = 96 795, 2 505 events), I21 Acute myocardial infarction (light blue; n = 97 237, 1 701 events), I50 Heart failure (green; n = 97 185, 1 752 events), I63 Cerebral infarction (dark blue; n = 97 249, 1 056 events), I64 Stroke, not specified as haemorrhage or infarction (orange; n = 97 283, 270 events) and I73 Other peripheral vascular disease (lavender; n = 97 164, 985 events). The results are computed for a subset of UK biobank dataset excluding individuals with self-reported use of cholesterol lowering medication. The biomarkers represent 37 clinically validated biomarkers in the Nightingale NMR platform. Hazard ratios and 95% confidence intervals (CI) are shown per SD-scaled biomarker concentrations to facilitate comparison. The models were adjusted for age, sex and UK biobank assessment center, using age as the timescale of the Cox proportional hazards regression. Filled points indicate statistically significant ($p < 5e-5$) associations, hollow points non-significant ones. BCAA indicates branched-chain amino acids; DHA: docosahexaenoic acid; MUFA: monounsaturated fatty acids; PUFA: polyunsaturated fatty acids; SFA: saturated fatty acids.

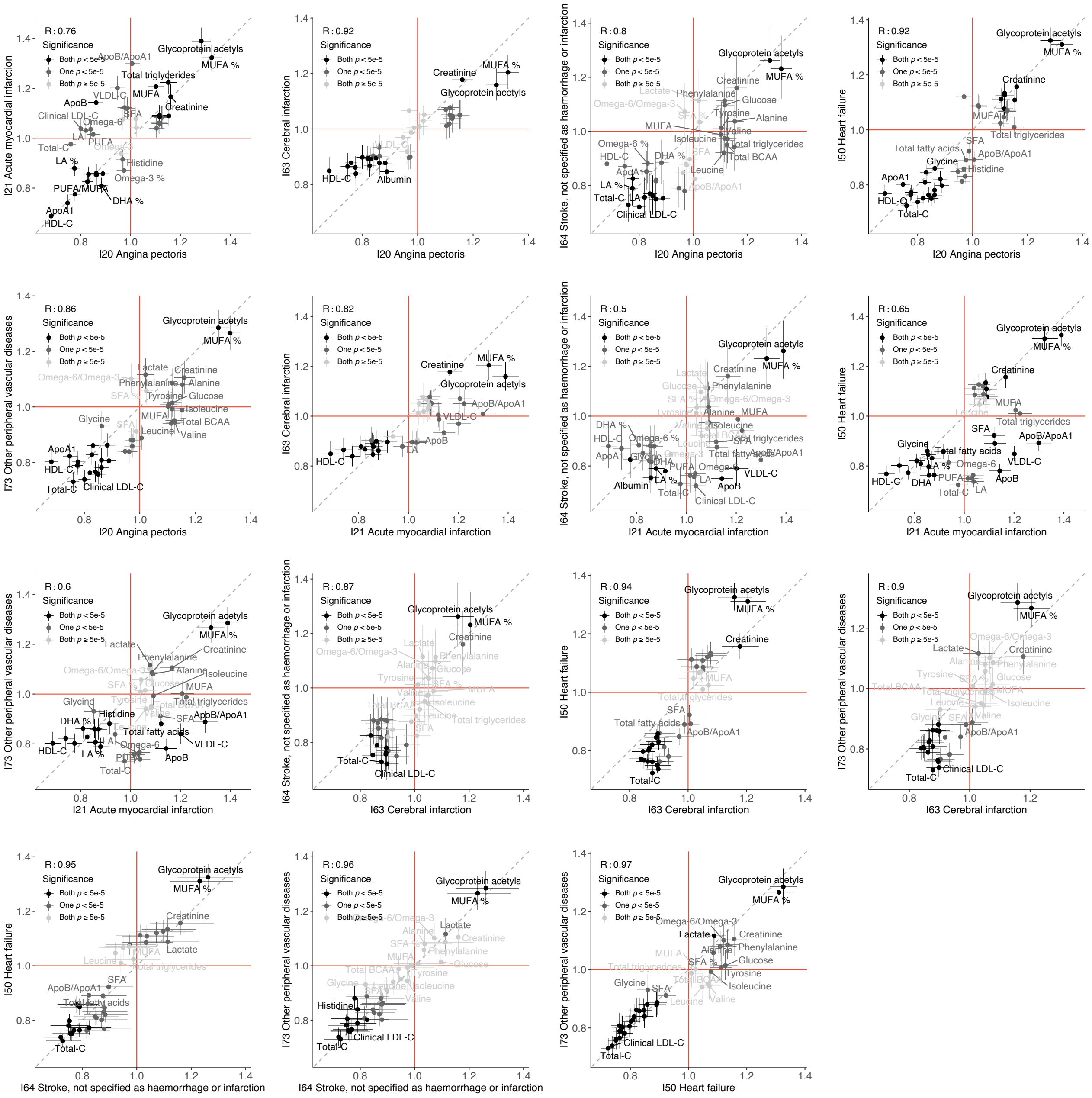

Supplementary Figure 13. Correlation of biomarker association signatures for the incidence of cardiovascular diseases. Scatterplots of the association signatures between pairs of different cardiovascular disease endpoints (I20 Angina pectoris (n = 115 154, 4 502 events), I21 Acute myocardial infarction (n = 116 781, 2 523 events)., I50 Heart failure (n = 117 498, 3 150 events), I63 Cerebral infarction (n = 117 724, 1 608 events), I64 Stroke, not specified as haemorrhage or infarction (n = 117 865, 456 events) and I73 Other peripheral vascular disease (n = 117 597, 1 666 events)). The hazard ratios for each biomarker (points) are given with 95% confidence intervals (CI) in vertical and horizontal error bars. The models were adjusted for age, sex and UK biobank assessment center, using age as the timescale of the Cox proportional hazards regression. The coloring of the points indicates the significance of the biomarker association for the pair of diseases. The red lines denote a hazard ratio of one, and the grey line denotes the diagonal. BCAA indicates branched-chain amino acids; DHA: docosahexaenoic acid; MUFA: monounsaturated fatty acids; PUFA: polyunsaturated fatty acids; SFA: saturated fatty acids.

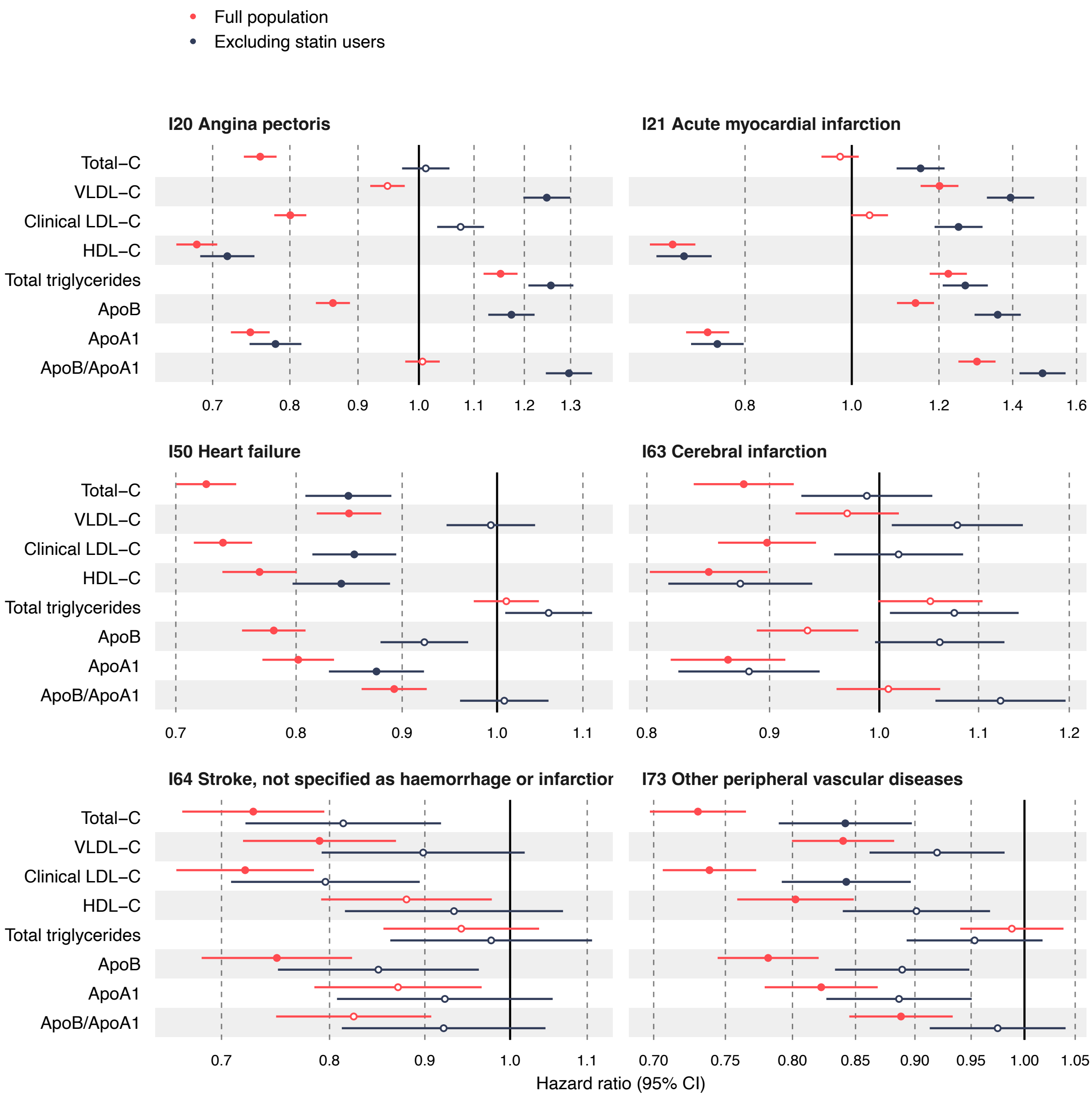

Supplementary Figure 14. Influence of lipid lowering medication on associations with cardiovascular diseases. Hazard ratios for cholesterol and other lipid-related measures against the incidence of six cardiovascular diseases in the full UK biobank dataset (red) and a subset excluding individuals with self-reported use of cholesterol lowering medication (blue). Hazard ratios and 95% confidence intervals (CI) are shown per SD-scaled biomarker concentrations to facilitate comparison. The models were adjusted for age, sex and UK biobank assessment center, using age as the timescale of the Cox proportional hazards regression. Filled points indicate statistically significant associations ($p < 5e-5$), and hollow points non-significant ones.

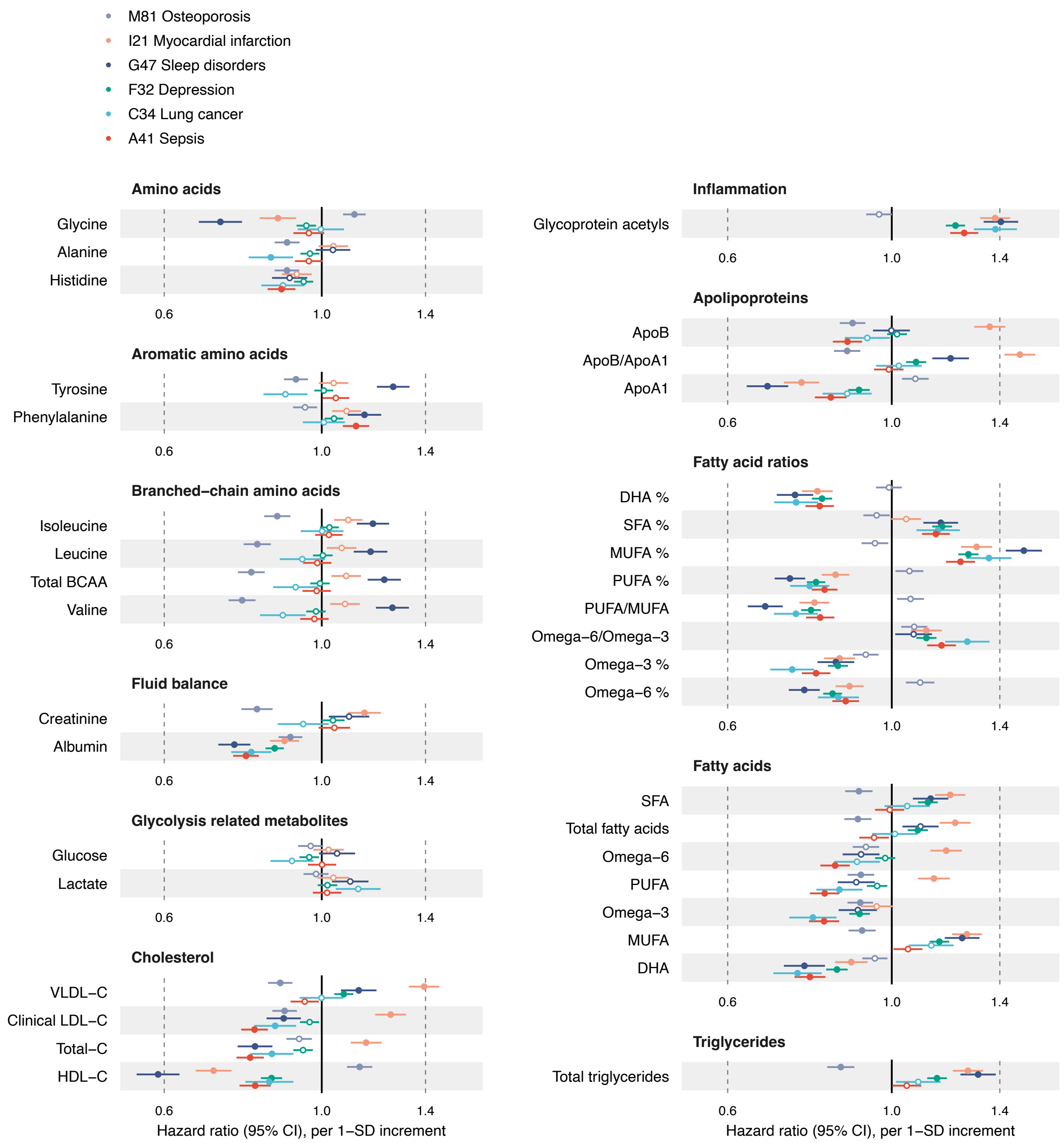

Supplementary Figure 15. Biomarker profiles for various types of diseases in a population free of cholesterol lowering medication. Hazard ratios of biomarkers with the incidence of 6 selected example diseases from distinct ICD-10 chapters, computed for a subset of UK biobank dataset excluding individuals with self-reported use of cholesterol lowering medication: A41 Sepsis (red; n = 97 164, 1 998 events), C34 Lung cancer (light blue; n = 97 262, 828 events), F32 Depression (green; n = 96 572, 4 173 events), G47 Sleep disorders (dark blue; n = 96 901, 1 225 events), I21 Myocardial infarction (orange; n = 97 237, 1 701 events) and M81 Osteoporosis (lavender; n = 96 947, 2 576 events). The biomarkers represent 37 biomarkers that are clinically validated in the Nightingale NMR platform. Hazard ratios and 95% confidence intervals (CI) are shown per SD-scaled biomarker concentrations to facilitate comparison. The models were adjusted for age, sex and UK biobank assessment center, using age as the timescale of the Cox proportional hazards regression. Filled points indicate statistically significant ($p < 5e\text{-}5$) associations, hollow points non-significant ones. BCAA indicates branched-chain amino acids; DHA: docosahexaenoic acid; MUFA: monounsaturated fatty acids; PUFA: polyunsaturated fatty acids; SFA: saturated fatty acids.

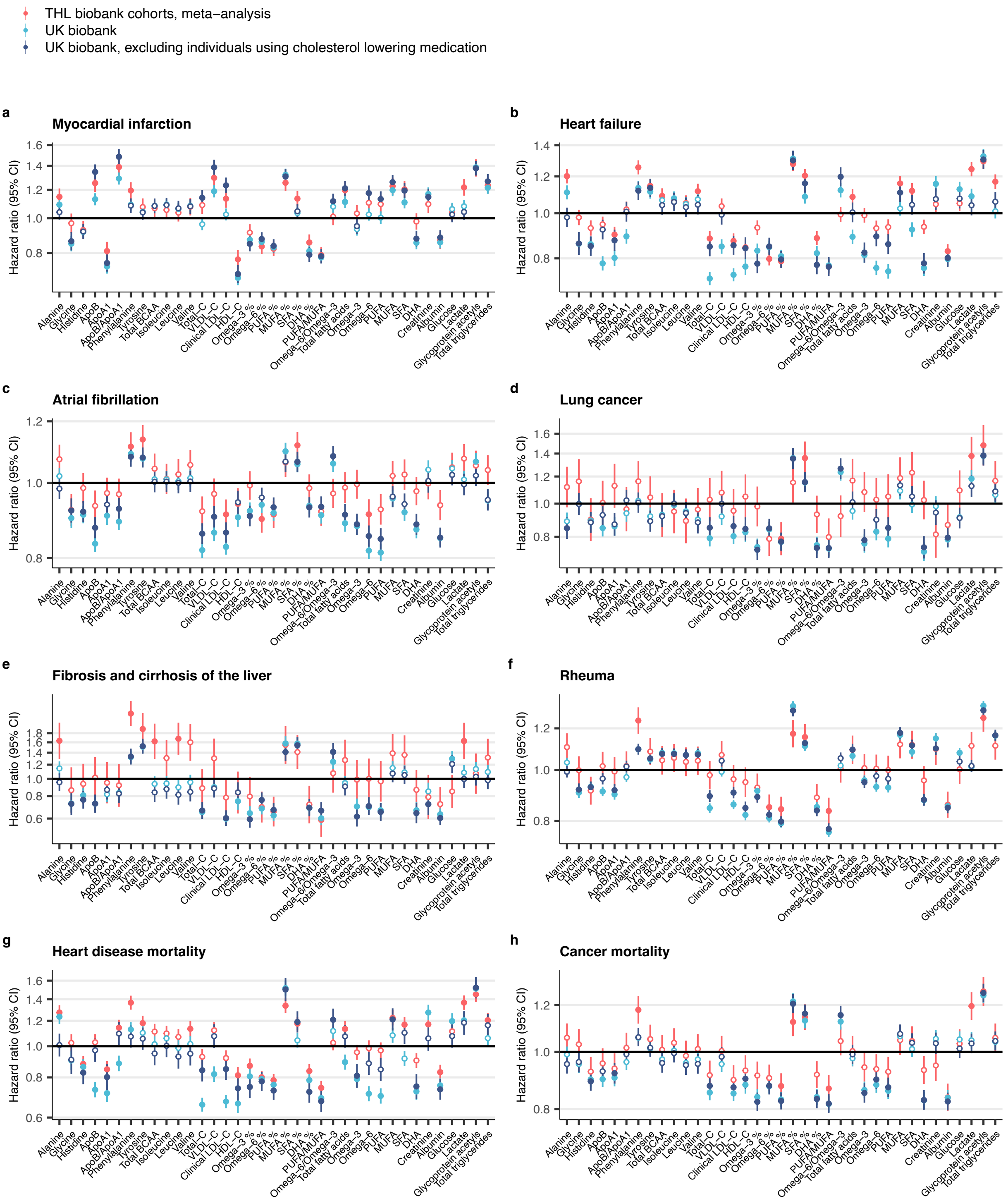

Supplementary Figure 16. Replication of biomarker associations. Replication of biomarker associations across eight remaining overlapping endpoints in THL Biobank (red) and UK Biobank for the full study population (light blue) as well as for individuals without self-reported use of cholesterol-lowering medication (dark blue): a) Myocardial infarction, b) Heart failure, c) Atrial fibrillation, d) Lung cancer, e) Fibrosis and cirrhosis of the liver, f) Rheuma, g) Heart disease mortality and h) Cancer mortality. Results from THL biobank represent meta-analyzed results for individuals from 5 prospective Finnish cohorts (FINRISK 1997, 2002, 2007, and 2012, and Health 2000). Hazard ratios (HRs) and 95% confidence intervals (CI) are shown per SD-scaled biomarker concentrations to facilitate comparison across the biomarkers. The models were adjusted for age, sex and UK biobank assessment center, using age as the timescale of the Cox proportional hazards regression. Filled points indicate statistically significant associations ($p < 5e\text{-}5$), and hollow points non-significant ones. The black horizontal line denotes a hazard ratio of 1. Sample size and event numbers are shown in Supplementary Table 1. BCAA indicates branched-chain amino acids; DHA: docosahexaenoic acid; MUFA: monounsaturated fatty acids; PUFA: polyunsaturated fatty acids; SFA: saturated fatty acids.

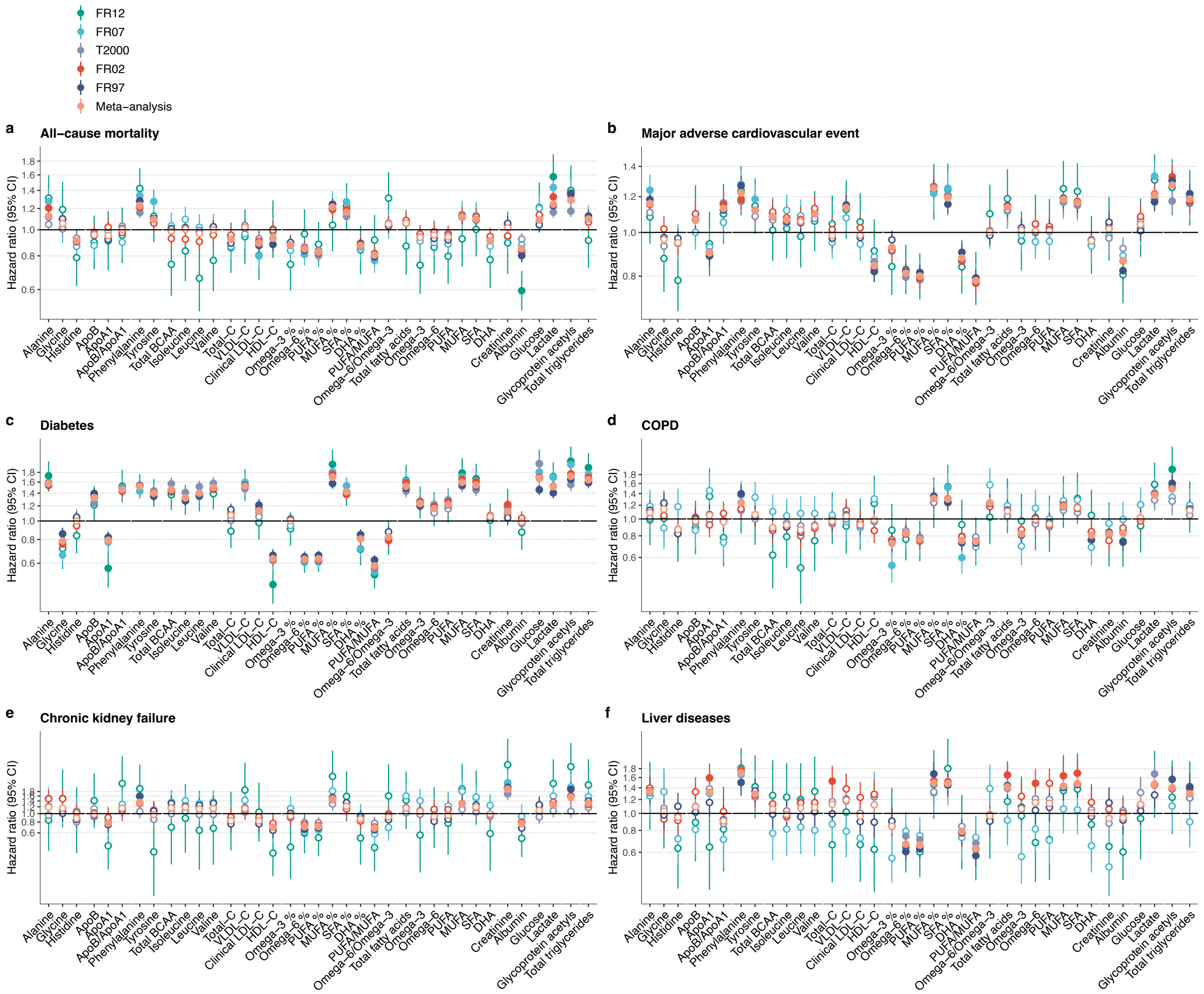

Supplementary Figure 17. Replication of biomarkers in each cohort. Replication of the biomarker associations across six endpoints by each cohort in Finnish Institute for Health and Welfare (THL) biobank: a) All-cause mortality, b) Major adverse cardiovascular event, c) Diabetes, d) Chronic obstructive pulmonary disease (COPD), e) Chronic kidney failure and f) Liver diseases. Results come from separate analyses of the 5 prospective Finnish cohorts (FINRISK 1997 (dark blue), 2002 (red), 2007 (light blue), and 2012 (green), and Health 2000 (lavender)) and a meta-analysis of these results (orange). Hazard ratios (HRs) and 95% confidence intervals (CI) are shown per SD-scaled biomarker concentrations to facilitate comparison across the biomarkers. The models were adjusted for age, sex and UK biobank assessment center, using age as the timescale of the Cox proportional hazards regression. Filled points indicate statistically significant associations ($p < 5e-5$), and hollow points non-significant ones. The black horizontal line denotes a hazard ratio of 1. The biomarkers represent 37 biomarkers that are clinically validated in the Nightingale NMR platform. Sample size and event numbers are shown in Supplementary Table 1. BCAA indicates branched-chain amino acids; DHA: docosahexaenoic acid; MUFA: monounsaturated fatty acids; PUFA: polyunsaturated fatty acids; SFA: saturated fatty acids.

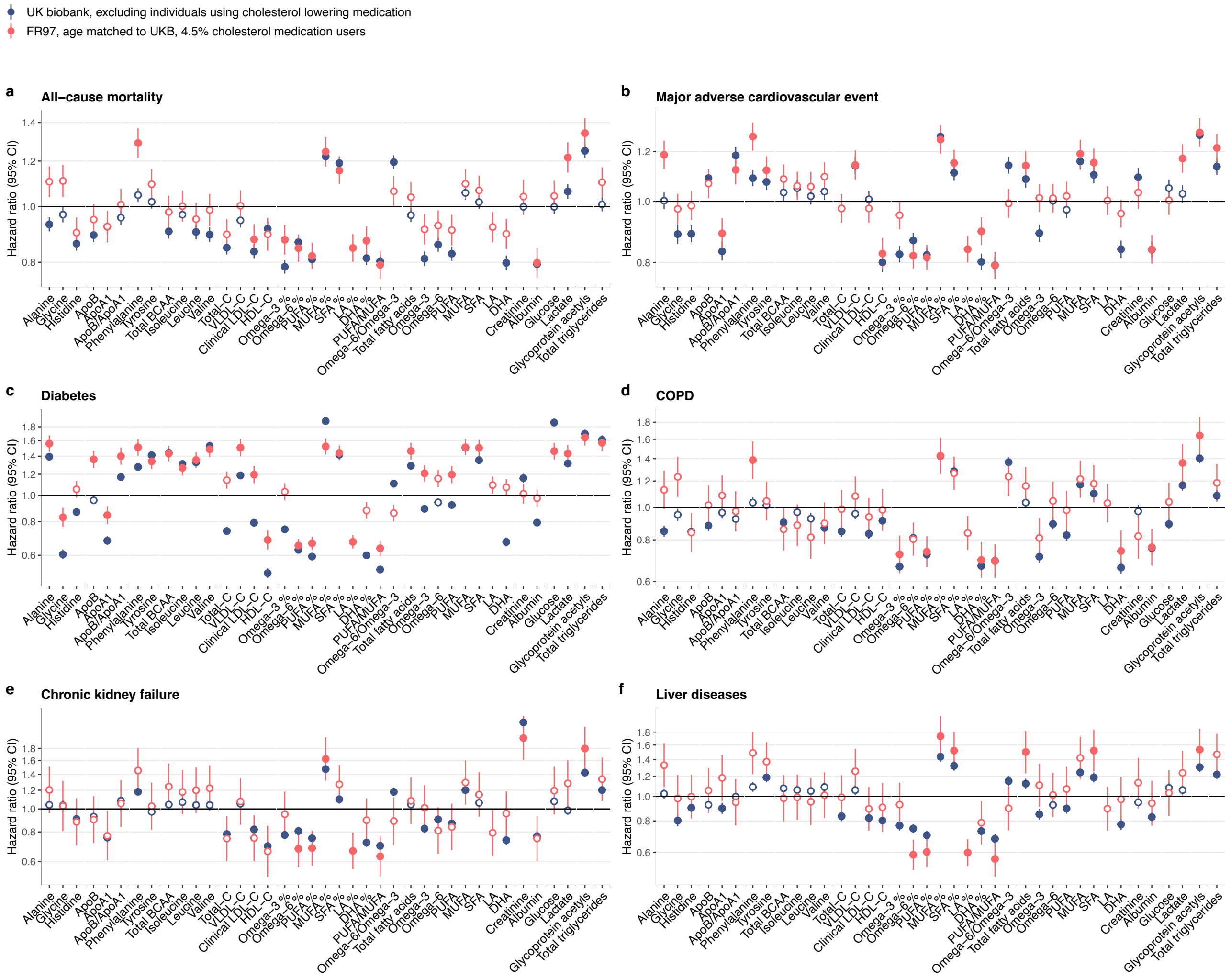

Supplementary Figure 18. Replication in FINRISK 1997 cohort. Replication of the biomarker associations across six endpoints in FINRISK 1997 cohort (in red), after matching the participant age to the age range in UK Biobank (in dark blue): a) All-cause mortality, b) Major adverse cardiovascular event, c) Diabetes, d) Chronic obstructive pulmonary disease (COPD), e) Chronic kidney failure and f) Liver diseases. For comparison, the associations are shown in UK Biobank after excluding individuals using cholesterol lowering medication. Hazard ratios (HRs) and 95% confidence intervals (CI) are shown per SD-scaled biomarker concentrations to facilitate comparison across the biomarkers. The black horizontal line denotes a hazard ratio of 1. The models were adjusted for age, sex and UK biobank assessment center, using age as the timescale of the Cox proportional hazards regression. Filled points indicate statistically significant associations ($p < 5e-5$), and hollow points non-significant ones. The biomarkers represent 37 biomarkers that are clinically validated in the Nightingale NMR platform. Sample size and event numbers are shown in Supplementary Table 1. BCAA indicates branched-chain amino acids; DHA: docosahexaenoic acid; MUFA: monounsaturated fatty acids; PUFA: polyunsaturated fatty acids; SFA: saturated fatty acids.

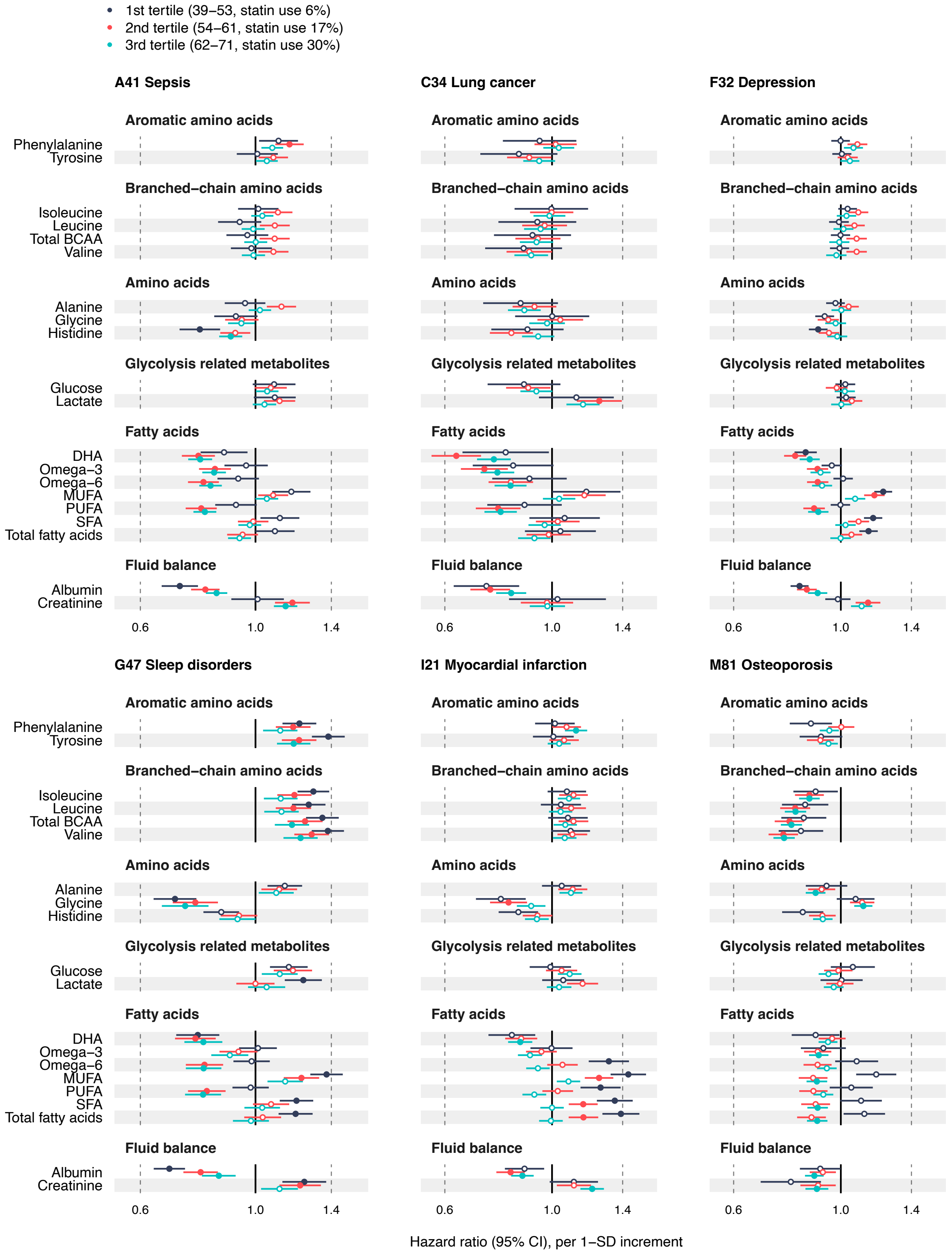

Supplementary Figure 19: Age-stratified biomarker profiles. Biomarker profiles stratified by age tertiles: 1st tertile (39-53 years of age; dark blue), 2nd tertile (54-61 years of age; red) and 3rd tertile (62-71 years of age; green). Results are shown for 20 biomarkers across six disease examples: A41 Sepsis (n = 117 806, 2 986 events), C34 Lung cancer (n = 117 964, 1 210 events), F32 Depression (n = 116 993, 5 455 events), G47 Sleep disorders (n = 117 325, 1 865 events), I21 Myocardial infarction (n = 116 797, 2 523 events) and M81 Osteoporosis (n = 117 538, 3 326 events). Hazard ratios and 95% confidence intervals (CI) are shown per SD. The models were adjusted for age, sex and UK biobank assessment center, using age as the timescale of the Cox proportional hazards regression. Filled points indicate statistically significant associations ($p < 5e-5$), and hollow points non-significant ones. Similar forest plots for all 249 NMR biomarkers across all endpoints analysed are provided in the biomarker-disease atlas webtool. BCAA indicates branched-chain amino acids; DHA: docosahexaenoic acid; MUFA: monounsaturated fatty acids; PUFA: polyunsaturated fatty acids; SFA: saturated fatty acids.
