# Supplementary material for: Atlas of plasma NMR biomarkers for health and disease in 118,461 individuals from the UK Biobank
Source: Nat Commun. 2023 Feb 3;14:604. doi: 10.1038/s41467-023-36231-7 (PMC9898515; doi:10.1038/s41467-023-36231-7)
Supplement: Supplementary file 3 — Reporting Summary [file 41467_2023_36231_MOESM3_ESM.pdf]

Corresponding author(s): Peter Würtz

Last updated by author(s): Dec 22, 2022

Reporting Summary

Nature Portfolio wishes to improve the reproducibility of the work that we publish. This form provides structure for consistency and transparency in reporting. For further information on Nature Portfolio policies, see our Editorial Policies and the Editorial Policy Checklist.

Statistics

For all statistical analyses, confirm that the following items are present in the figure legend, table legend, main text, or Methods section.

n/a	Confirmed	
☐	☒	The exact sample size (n) for each experimental group/condition, given as a discrete number and unit of measurement
☐	☒	A statement on whether measurements were taken from distinct samples or whether the same sample was measured repeatedly
☐	☒	The statistical test(s) used AND whether they are one- or two-sided<br>*Only common tests should be described solely by name; describe more complex techniques in the Methods section.*
☐	☒	A description of all covariates tested
☐	☒	A description of any assumptions or corrections, such as tests of normality and adjustment for multiple comparisons
☐	☒	A full description of the statistical parameters including central tendency (e.g. means) or other basic estimates (e.g. regression coefficient) AND variation (e.g. standard deviation) or associated estimates of uncertainty (e.g. confidence intervals)
☐	☒	For null hypothesis testing, the test statistic (e.g. F, t, r) with confidence intervals, effect sizes, degrees of freedom and P value noted<br>*Give P values as exact values whenever suitable.*
☒	☐	For Bayesian analysis, information on the choice of priors and Markov chain Monte Carlo settings
☒	☐	For hierarchical and complex designs, identification of the appropriate level for tests and full reporting of outcomes
☐	☒	Estimates of effect sizes (e.g. Cohen's d, Pearson's r), indicating how they were calculated

Our web collection on statistics for biologists contains articles on many of the points above.

Software and code

Policy information about availability of computer code

Data collection	Plasma biomarkers were measured by Nightingale Health Biomarker Analysis Platform, quantification version 4.0.1 (2020, proprietary software). This includes automated spectral processing.
Data analysis	Data analyses on biomarkers and clinical data from UK Biobank were conducted using R statistical software (completed and tested with version 4.1.1).

For manuscripts utilizing custom algorithms or software that are central to the research but not yet described in published literature, software must be made available to editors and reviewers. We strongly encourage code deposition in a community repository (e.g. GitHub). See the Nature Portfolio guidelines for submitting code & software for further information.

Data

Policy information about availability of data

All manuscripts must include a data availability statement. This statement should provide the following information, where applicable:

- Accession codes, unique identifiers, or web links for publicly available datasets
- A description of any restrictions on data availability
- For clinical datasets or third party data, please ensure that the statement adheres to our policy

The Nightingale Health NMR biomarker data have been released to the UK Biobank resource in spring 2021 (https://biobank.ndph.ox.ac.uk/showcase/label.cgi?id=220). The UK Biobank data are available for approved researchers through the UK Biobank data-access protocol. Data from FINRISK and Health 2000 cohorts can be accessed through THL Biobank (https://thl.fi/en/web/thl-biobank). We provide access to all biomarker-disease summary statistics for academic use through an

interactive webtool biomarker-atlas.nightingale.cloud. Source data are provided with this paper.

Human research participants

Policy information about studies involving human research participants and Sex and Gender in Research.

Reporting on sex and gender	Analyses are adjusted for sex and sex-stratified analyses reported for sex-specific diseases (148 female diseases, 18 male diseases; Supplementary Table 3).
Population characteristics	Table 1 shows characteristics of the participants with NMR biomarker data currently available in the UK Biobank. The EDTA plasma samples were picked randomly and are therefore representative of the 502,543 participants in the full cohort.
Recruitment	Recruitment of study participants in the UK Biobank is based on volunteer enrollment. Participants are therefore on average more healthy than the general population. This results in lower disease rates than in the general population, but association magnitudes of biomarkers with incident disease are expected to be minimally influenced by this.
Ethics oversight	The UK Biobank study was approved by the North West Multi-Centre Research Ethics Committee and all participants provided written informed consent. The study protocol is available online (https://www.ukbiobank.ac.uk).

Note that full information on the approval of the study protocol must also be provided in the manuscript.

Field-specific reporting

Please select the one below that is the best fit for your research. If you are not sure, read the appropriate sections before making your selection.

☒ Life sciences ☐ Behavioural & social sciences ☐ Ecological, evolutionary & environmental sciences

For a reference copy of the document with all sections, see nature.com/documents/nr-reporting-summary-flat.pdf

Life sciences study design

All studies must disclose on these points even when the disclosure is negative.

Sample size	The UK Biobank is a cohort of 500,000 individuals. A random subset of around 120,000 samples were profiled by Nightingale Health's NMR biomarker profiling platform in the first phase of the project. No specific procedure to determine the sample size was applied before the analyses. This sample size was considered sufficient, as it is over 10-fold larger than most prior studies on metabolic biomarkers.
Data exclusions	Biomarker values outside four interquartile ranges from median were considered outliers and excluded from the analyses. For analyses of future incidence of a given disease, individuals with the same prevalent disease were excluded in order to remove samples with an already existing clinical presentation of the disease.
Replication	The technical repeatability of the biomarker measurements was tested by blind duplicate samples as described in Supplementary Methods. The biomarker associations measured by NMR were validated with those measured by clinical chemistry in case where overlapping biomarkers were available in the UK Biobank (8 overlapping biomarkers). The biomarker associations were further replicated in a meta-analysis of five independent cohorts from THL biobank for 14 overlapping endpoints.
Randomization	Analyses were adjusted for age, sex and UK Biobank assessment centre.
Blinding	Measurements of the metabolic biomarkers were conducted blinded prior to the linkage to the UK Biobank health outcomes.

Reporting for specific materials, systems and methods

We require information from authors about some types of materials, experimental systems and methods used in many studies. Here, indicate whether each material, system or method listed is relevant to your study. If you are not sure if a list item applies to your research, read the appropriate section before selecting a response.

March 2021

Materials & experimental systems

n/a	Involved in the study
☒	☐ Antibodies
☒	☐ Eukaryotic cell lines
☒	☐ Palaeontology and archaeology
☒	☐ Animals and other organisms
☒	☐ Clinical data
☒	☐ Dual use research of concern

Methods

n/a	Involved in the study
☒	☐ ChIP-seq
☒	☐ Flow cytometry
☒	☐ MRI-based neuroimaging
